# Development of an ultrasensitive microfluidic assay for the analysis of Glial fibrillary acidic protein (GFAP) in blood

**DOI:** 10.3389/fmolb.2023.1175230

**Published:** 2023-04-24

**Authors:** Badrieh Fazeli, André Huss, Nerea Gómez de San José, Markus Otto, Hayrettin Tumani, Steffen Halbgebauer

**Affiliations:** ^1^ Department of Neurology, Ulm University Hospital, Ulm, Germany; ^2^ German Center for Neurodegenerative Diseases (DZNE e.V.), Ulm, Germany; ^3^ Department of Neurology, Martin-Luther-University Halle-Wittenberg, Halle, Germany

**Keywords:** GFAP, biomarker, blood, CSF, microfluidic assay, multiple sclerosis, Alzheimer’s disease

## Abstract

**Introduction:** A rapid and reliable detection of glial fibrillary acidic protein (GFAP) in biological samples could assist in the diagnostic evaluation of neurodegenerative disorders. Sensitive assays applicable in the routine setting are needed to validate the existing GFAP tests. This study aimed to develop a highly sensitive and clinically applicable microfluidic immunoassay for the measurement of GFAP in blood.

**Methods:** A microfluidic GFAP assay was developed and validated regarding its performance. Subsequently, serum and cerebrospinal fluid (CSF) of Alzheimer’s disease (AD), Multiple Sclerosis (MS) and control patients were analyzed with the established assay, and levels were compared to the commercial GFAP Simoa discovery kit.

**Results:** The developed GFAP assay showed a good performance with a recovery of 85% of spiked GFAP in serum and assay variations below 15%. The established assay was highly sensitive with a calculated lower limit of quantification and detection of 7.21 pg/mL and 2.37 pg/mL, respectively. GFAP levels were significantly increased in AD compared to control patients with advanced age (*p* = 0.002). However, GFAP levels revealed no significant increase in MS compared to control patients in the same age range (*p* = 0.140). Furthermore, serum GFAP levels evaluated with the novel microfluidic assay strongly correlated with Simoa concentrations (*r* = 0.88 (95% CI: 0.81–0.93), *p* < 0.0001).

**Conclusion:** We successfully developed a sensitive and easy-to-use microfluidic assay to measure GFAP in blood. Furthermore, we could confirm previous findings of elevated GFAP levels in AD by applying the assay in a cohort of clinically characterized patients.

## Introduction

An accurate and early diagnosis of neurodegenerative diseases is crucial for successful treatment. Recent biological approaches focus on the analysis of protein biomarkers in cerebrospinal fluid (CSF) or blood to improve individualized therapy and evaluate therapy outcomes (Koníčková et al., 2022). A promising biomarker will need to not only demonstrate the onset of the disease, but also the disease severity and treatment efficacy (Hansson, 2021). Several studies have suggested potential biomarkers for neurodegenerative diseases like Alzheimer’s disease (AD) (Blennow and Zetterberg, 2018; Valenza and Scuderi, 2022) or inflammatory diseases like Multiple Sclerosis (MS) (Paul et al., 2019; Yang et al., 2022).

Glial fibrillary acidic protein (GFAP), a type-III intermediate filament, is a cell-specific marker that distinguishes astrocytes from other glial cells during the development of the central nervous system (CNS). Human GFAP is a 432-amino acid long polypeptide of 55 kDa encoded by the GFAP gene on chromosome 17q21 (Messing and Brenner, 2020). Since the first report of GFAP, ten isoforms resulting from alternative splicing were identified, of which GFAPα is the canonical isoform (Kim et al., 2022).

A growing body of evidence recognized GFAP and its elevated expression as a universal marker for reactive astrogliosis (Petzold et al., 2002; Sofroniew and Vinters, 2010; Zamanian et al., 2012) and astrocytic damages (Yang et al., 2019). The potential of GFAP in clinical diagnosis was proved in several multi-center studies when a GFAP and ubiquitin carboxy-terminal hydrolase L1 blood test was authorized by the FDA to be used in mild traumatic brain injury (Petzold, 2015; Wang et al., 2018; Czeiter et al., 2020; Yuh et al., 2021). In addition, blood GFAP concentrations can also be used as an accessible biomarker for AD pathology (O’Connor et al., 2022), in the differential diagnosis of AD as well as the conversion of MCI-to-dementia in AD cohorts (Cicognola et al., 2021; Oeckl et al., 2022). Furthermore, GFAP is recognized as a marker for MS severity (Abdelhak et al., 2018; Högel et al., 2018; Abdelhak et al., 2019; Barro et al., 2023) and blood GFAP could be a promising biomarker to distinguish between classic autoimmune inflammatory astrocytopathy, neuromyelitis optica spectrum disorders (NMOSD) and MS (Storoni et al., 2012; Kim et al., 2022).

Several sensitive methods for GFAP detection in CSF are commercially available. However, the detection of GFAP in blood has historically been a challenge due to its significantly lower blood than CSF levels. Even though developing highly sensitive assays, like single-molecule arrays (Simoa), has enabled the detection and measurement of serum GFAP in discovery kits, novel, sensitive, and easily transferable diagnostic tests confirming Simoa blood assay findings in the literature are needed.

This study reports on the development and validation of a novel, highly sensitive, and easy-to-use immunoassay to measure serum and CSF GFAP using a microfluidic system. We applied the novel assay to analyze GFAP concentrations in three diagnostic groups including AD, MS, and control patients (Con). The cohort was also measured with Simoa and the correlation between the assays was determined.

## Materials and method

### Patient selection

98 CSF and serum samples from three diagnostic groups (AD, MS, Con) were examined in this study. The samples were collected in the department of Neurology of Ulm University Hospital between 2014 and 2020. All patients or their legal proxies were informed and signed the consent for inclusion in this study. The study was approved by the local Ethics committee from the University of Ulm (approval number: 20/10) and conducted following the Declaration of Helsinki. To analyze serum GFAP, samples of AD (*n* = 18), MS (*n* = 17), and Con (*n* = 40) were collected. Furthermore, GFAP CSF was measured in AD (*n* = 13) and Con (*n* = 10) samples. The diagnosis of AD and MS patients was revised according to the International Working Group 2 criteria (Dubois et al., 2014) and the 2017 revision of the McDonald criteria (Thompson et al., 2018), respectively. MS patients included 11 patients in the relapsing-remitting MS (RRMS) stage and 6 patients in the primary progressive MS (PPMS)/Secondary progressive MS (SPMS) stages. 40 Con patients admitted for ruling out neurological disease (presenting with tension-type headache, temporary sensory symptoms, dizziness) showed no clinical and radiological signs of neurodegenerative or inflammatory disease. All Con patients underwent a lumbar puncture to eliminate an acute or chronic inflammation (incl. Normal leukocyte count, intact blood-CSF barrier function (i.e., normal Albumin CSF-serum ratio), and absence of intrathecal immunoglobulin synthesis (incl. Quantitative analysis of IgG, IgA, IgM, and oligoclonal IgG bands) of the central nervous system. We split the control group into young controls (23–69 years old) which we used for comparison with the MS group (MS controls) and an AD control group consisting of Con patients with advanced age (63–88 years old).

### CSF and serum sampling

Serum was extracted from venous blood samples by centrifugation at 2000 *g* for 10 min and stored within 30 min at −80°C (Teunissen et al., 2009). CSF samples collected through lumbar puncture were centrifuged at 2000 *g* for 10 min and supernatants were aliquoted and frozen at −80°C within 30 min. GFAP serum and CSF levels were analyzed with a newly established microfluidic assay. Disease groups were randomized during measurements and serum and CSF quality control (QC) samples were included in duplicate in all runs. To monitor assay performance, the intra-assay coefficient of variation (CV) was assessed by measuring serum and CSF QC samples in ten replicates. The inter-assay reproducibility was determined by analysis of 5 replicates of serum and CSF QC samples in two different runs. The lower limit of quantification (LLOQ) was calculated as reported by Andreasson and colleagues (Andreasson et al., 2015). The limit of detection (LOD) was determined based on a signal of 3 SDs above the mean of 16 blanks. 90% of analyzed samples were between the LLOQ and upper LOQ. Samples below the LOD were set as LOD divided by 2. The GFAP recovery rate was assessed by spiking dilution series of two serum samples and one CSF sample with GFAP recombinant protein and results were calculated in percentage.

### Antibodies and recombinant protein

The novel immunoassay included a rabbit polyclonal antibody (16825-1-AP, Proteintech, Munich, Germany) against GFAP as capture and a recombinant rabbit monoclonal bovine serum albumin and azide-free antibody (EPR19996, Abcam, Cambridge, United Kingdom) as detector. The detector antibody was biotinylated in the ratio of biotin to antibody 40:1 according to the biotinylation protocol provided by Quanterix Corporation (Lexington, Massachusetts, United States). The capture antibody was conjugated with Digoxigenin-NHS according to Bio-techne (Minneapolis, United States) protocol. Recombinant GFAP (Ag10423) was purchased from Proteintech (Munich, Germany).

### Novel GFAP Ella assay for serum and CSF analysis

GFAP was measured with the Bio-techne microfluidic Ella platform using the newly established assay. The Ella platform is an automated, microfluidic method for the quantitative measurement of proteins in biological samples. The immunoassay operation takes place in small glass nano reactors (GNR) which are pre-coated with anti-digoxigenin antibodies, therefore digoxigenin-conjugated capture antibodies can be attached to the GNR wall. After samples ran through the channels binding to the capture antibody wash steps remove unbound analytes and the biotin-conjugated detector antibody completes the sandwich combination. Subsequently, a streptavidin-conjugated fluorophore produces detectable signals. All samples are automatically analyzed as triplicates.

Serum and CSF samples were diluted with sample diluent SD13 (Bio-techne, Minneapolis, United States) with dilution factors of 1:2 and 1:8, respectively. To assess the potential of GFAP measurement in plasma, 10 serum and plasma paired samples, both with dilution factors of 1:2, were also included. A series of calibrators with the known concentration of recombinant protein in the sample diluent buffer (SD13) was run in each cartridge. A working concentration of 3.5 μg/mL of the capture and detector antibodies was prepared using Reagent Diluent (Bio-techne, Minneapolis, United States). A final volume of 50 µl of either samples or antibodies was dispensed into related wells on a 48-Digoxigenin Cartridge (Bio-techne, Minneapolis, United States) Finally, 1 mL of wash buffer (Bio-techne, Minneapolis, United States) was added to designated wells.

### Simoa measurement for serum analysis

For Simoa analysis serum samples were measured with the Quanterix HD-X analyzer using the Simoa GFAP Discovery kit according to the manufacturer’s instructions (Quanterix, Massachusetts, United States).

### Statistical methods

Statistical analyses were performed with GraphPad Prism V.8.3.0 (GraphPad Software, La Jolla, CA, United States). The Spearman rank correlation coefficient was applied to determine correlations between Simoa and the novel Ella assay. The Mann-Whitney U-test was used to compare each patient group with the corresponding control group. For all analyses, *p* < 0.05 was considered statistically significant.

## Results

### Performance of the established Ella assay for the detection of GFAP

The Ella assay covered a range from 3.7 to 2,700 pg/mL. The assay reproducibility was proved based on measured inter-assay (14% for serum and 7% for CSF) and intra-assay (8% for serum and 6% for CSF) coefficient of variation. The LLOQ and LOD were determined to be 7.21 pg/mL and 2.37 pg/mL, respectively. Moreover, the dilution test results suggested the most effective dilution factors of 1:2 for serum and 1:8 for CSF samples. In addition, the recovery of spiked recombinant protein in the mentioned dilution factors was determined to be 85% and 97%, respectively. Finally, the GFAP stability for handling and storage conditions was assessed. The results suggested that GFAP in serum was stable at 4°C and RT for 3 days and five freeze-thaw cycles (FTCs). GFAP CSF levels deviated less than 20%, for up to 6 h at RT and 4°C and three FTCs. However, there was a trend to lower levels with increasing FTCs. No cross-reaction with human albumin or immunoglobulin G was observed. Statistical evaluations of the obtained results of paired serum and plasma samples indicated no significant differences. More details on the performance of the assay can be found in the Supplementary Materials.

### Clinical and demographic features

To validate our assay in a clinically characterized cohort, we evaluated 98 serum and CSF samples including serum samples of 18 AD, 17 MS, and 40 controls, as well as CSF samples of 13 AD and 10 controls. All relevant demographic and clinical parameters of the diagnostic groups are summarized in Table 1. AD controls were slightly older than AD patients (*p* = 0.0010), whereas there was no difference in age between the MS and respective control patients (*p* = 0.2444). A correlation between age and GFAP concentration was observed in the control cohort (*r* = 0.65 (95% CI: 0.41–0.80), *p* < 0.0001).

**TABLE 1:** Demographic and clinical parameters of the diagnostic groups.

Group	MS		AD		AD	
	Patients	Control	Patient	Control	Patient	Control
Number	17	20	18	20	13	10
Female (%)	80	40	66	55.5	69	80
Age (years)	28 (24.75–53.75)	38.5 (30–55.25)	69.5 (61.25–79)	75.5 (70.25–82)	68 (59.50–76.50)	78 (73.25–86.25)
Sample type	Serum	Serum	Serum	Serum	CSF	CSF
GFAP concentration (pg/mL) by Ella	11.88 (4.55–16.56)	7.64 (3.01–11.72)	29.20 (22.05–37.25)	15.23 (9.97–21.15)	1,304 (1,204–2,812)	1,000 (721.6–1,242)
GFAP concentration (pg/mL) by Simoa	91.55 (68.23–151.4)	80.95 (59.53–113.4)	249.8 (162.9–296.4)	151.2 (121.4–233.8)	n.a.	n.a.

All given values are median with the interquartile range in brackets. (25–75 percentile). AD, Alzheimer’s disease; CSF, Cerebrospinal fluid; GFAP, Glial fibrillary acidic protein; MS, multiple sclerosis; n. a, not applicable.

### GFAP levels in the diagnostic groups

**FIGURE 1:**
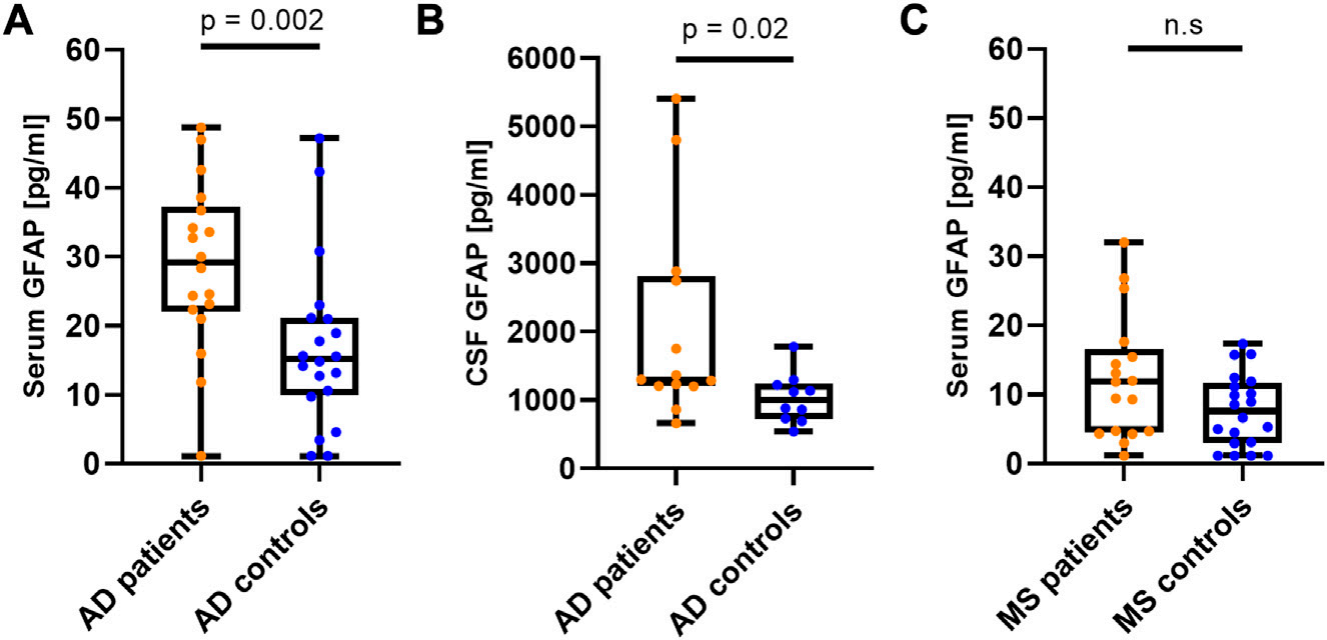
CSF and serum GFAP concentration result in diagnostic groups. Serum and CSF GFAP levels in patients with AD and MS compared to control patients applying the newly developed microfluidic assay. **(A)** Serum GFAP levels in AD. **(B)** CSF GFAP levels in AD. **(C)** Serum GFAP levels in MS. All GFAP concentrations are displayed as box plots with single patients as dots. Illustrated are the median concentration, the 25% and 75% percentile, and whiskers from minimum to maximum. AD, Alzheimer’s disease; GFAP, Glial fibrillary acidic protein; MS, multiple sclerosis; n. s, not significant.

GFAP levels were significantly increased in the AD compared to the control group in serum (*p* < 0.0029) (Figure 1A) and in CSF (*p* < 0.0222) (Figure 1B). In contrast, the increase of serum GFAP concentration in MS compared to Con was not significant (*p* = 0.1403) (Figure 1C).

### Correlation of serum GFAP concentrations

**FIGURE 2:**
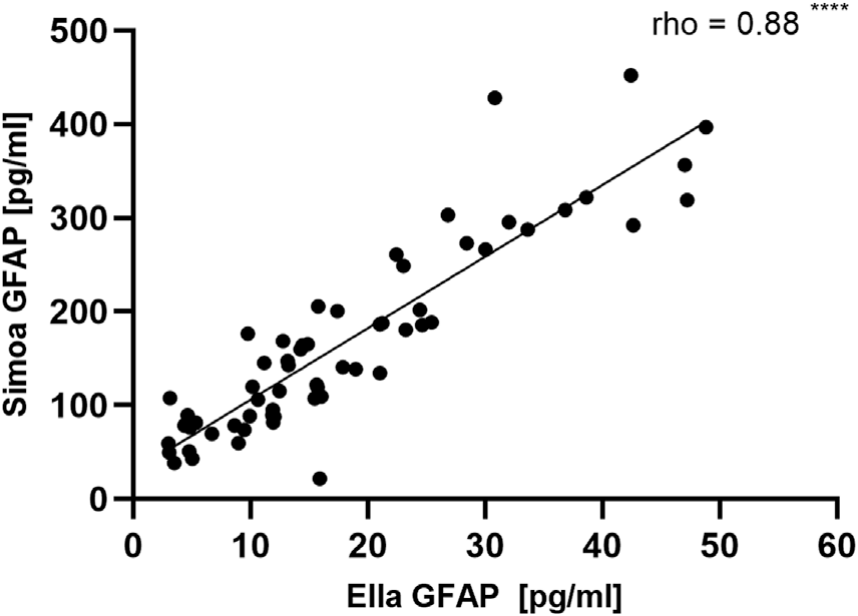
Correlation of measured GFAP values for serum samples by Ella and Simoa. 63 serum samples were analyzed with the novel Ella assay and the available Simoa GFAP discovery kit. The results correlated significantly between the two assays [*r* = 0.88 (95% CI: 0.81–0.93), *p* < 0.0001]. CI, Confidence interval; GFAP, Glial fibrillary acidic protein; Simoa, single molecule arrays.

To compare the newly established Ella with the Simoa assay, we measured the cohort with the GFAP Simoa discovery kit and correlation analyses were performed (Figure 2). Ella GFAP levels significantly correlated with related Simoa values (*r* = 0.88 (95% CI: 0.81–0.93), *p* < 0.0001).

## Discussion

This manuscript describes the establishment and validation of a promising, easy-to-handle Ella assay to detect GFAP quantitatively in serum and CSF for clinical routine analysis. The novel immunoassay yielded a good performance and obtained results that demonstrated strong specificity, reliability, and sensitivity. Furthermore, we validated the assay in a cohort of AD, MS, and non-degenerative disease control patients. The analysis of serum GFAP in our AD cohort revealed a significant increase compared to the non-neurodegenerative and non-inflammatory controls, which is in agreement with earlier studies (Oeckl et al., 2019; Oeckl et al., 2022; Benedet et al., 2021). We observed no significant increase in MS compared to control patients which could be related to the relapsing course of most of the MS patients. It was reported that GFAP could be a disease severity biomarker in MS and higher concentrations were observed in the progressive forms of the disease (Abdelhak et al., 2018). Indeed, our results confirm studies showing no elevation of serum GFAP in RRMS patients compared to control groups (Linker et al., 2009; Axelsson et al., 2011; Mahad et al., 2015; Mañé-Martínez et al., 2016; Abdelhak et al., 2017; Huss et al., 2020; Sun et al., 2021).

In agreement with previous studies (Abdelhak et al., 2018; Oeckl et al., 2019; Oeckl et al., 2022; Chatterjee et al., 2021), a positive association between GFAP levels and age was discovered using our newly established assay. It was suggested that elevated GFAP expression could be a direct response of astrocytes to the oxidative stress in aging (Morgan et al., 1997) or hormonal mechanisms of aging (Porchet et al., 2003).

Comparing serum and CSF GFAP variations, more significant differences in serum GFAP levels were obtained by comparing the diagnostic groups to the controls. This corroborates studies finding the same phenomenon. This might be explained by the probability of the direct drainage of GFAP into the bloodstream from astrocytic end feet which are part of the neurovascular unit (Abdelhak et al., 2018; Bolsewig et al., 2022). Our findings, therefore, confirm other studies arguing for a better diagnostic power of serum than CSF GFAP (Oeckl et al., 2019).

In our study, we found a high GFAP stability in serum and a trend of decreasing CSF GFAP values after several FTCs. Indeed, several recent studies have reported significantly higher serum GFAP stability compared to CSF after several FTCs as well as storage in RT or 2°C–8°C. These studies evaluated different neurodegenerative disease cohorts, and all showed that the GFAP concentration remains more consistent in serum regardless of FTC stage (up to four) or storage condition, whereas it decreases significantly after only two FTCs in CSF (Abdelhak et al., 2019; Ashton et al., 2021; Simrén et al., 2022; van Lierop et al., 2022; Verberk et al., 2022).

The strong correlation between the measured values with the novel Ella assay and the Simoa discovery kit confirmed the reliability of the established assay and the applicability of the novel assay in the measurement of clinical cohorts. However, the absolute values were lower in the Ella assay. This might be due to different capture and detector antibodies used in the two assays possibly recognizing different parts of GFAP. In addition, the comparison of the LLOQ and LOD of both methods revealed that the Simoa is slightly more sensitive than our homemade assay. The advantages of the new GFAP Ella assay are the higher GFAP recovery in serum compared to the Simoa assay, and the small benchtop platform used for analysis which is easy to use and highly applicable in clinical routine settings. Moreover, the Ella assay is more cost-effective compared to other blood GFAP detection methods and the measurement time for one cartridge is only around 75 min. On the other hand, the Ella open-cartridge format only fits 48 samples which need to be all analyzed in one run.

The strengths of our study are 1) the development of an extremely sensitive Ella assay for the analysis of GFAP in serum and CSF demonstrating a good performance regarding not only specificity and sensitivity but also robustness and reproducibility, 2) the validation of the assay in a clinical cohort and 3) the comparison of the assay with the commercial Simoa GFAP discovery kit. Limitations of the assay might be the higher LOD and LLOQ compared to Simoa and the small number of analyzed CSF samples.

In conclusion, we here present a novel and highly sensitive microfluidic immunoassay for the detection of GFAP in serum and CSF which is not only an alternative to existing methods but improves its applicability in clinical use. Moreover, we demonstrate a strong correlation with the Simoa method demonstrating the high reliability of the assay. These findings encourage the analysis of larger cohorts of patients, including other neurodegenerative and inflammatory diseases applying the novel assay.

## Data Availability

The original contributions presented in the study are included in the article/Supplementary Material, further inquiries can be directed to the corresponding author.
