# Supplementary material for: Development of an ultrasensitive microfluidic assay for the analysis of Glial fibrillary acidic protein (GFAP) in blood
Source: Front Mol Biosci. 2023 Apr 24;10:1175230. doi: 10.3389/fmolb.2023.1175230 (PMC10164994; doi:10.3389/fmolb.2023.1175230)
Supplement: Supplementary file 1 [file Table1.DOCX]

**Supplementary materials**

**Parallelism, Spike & recovery**


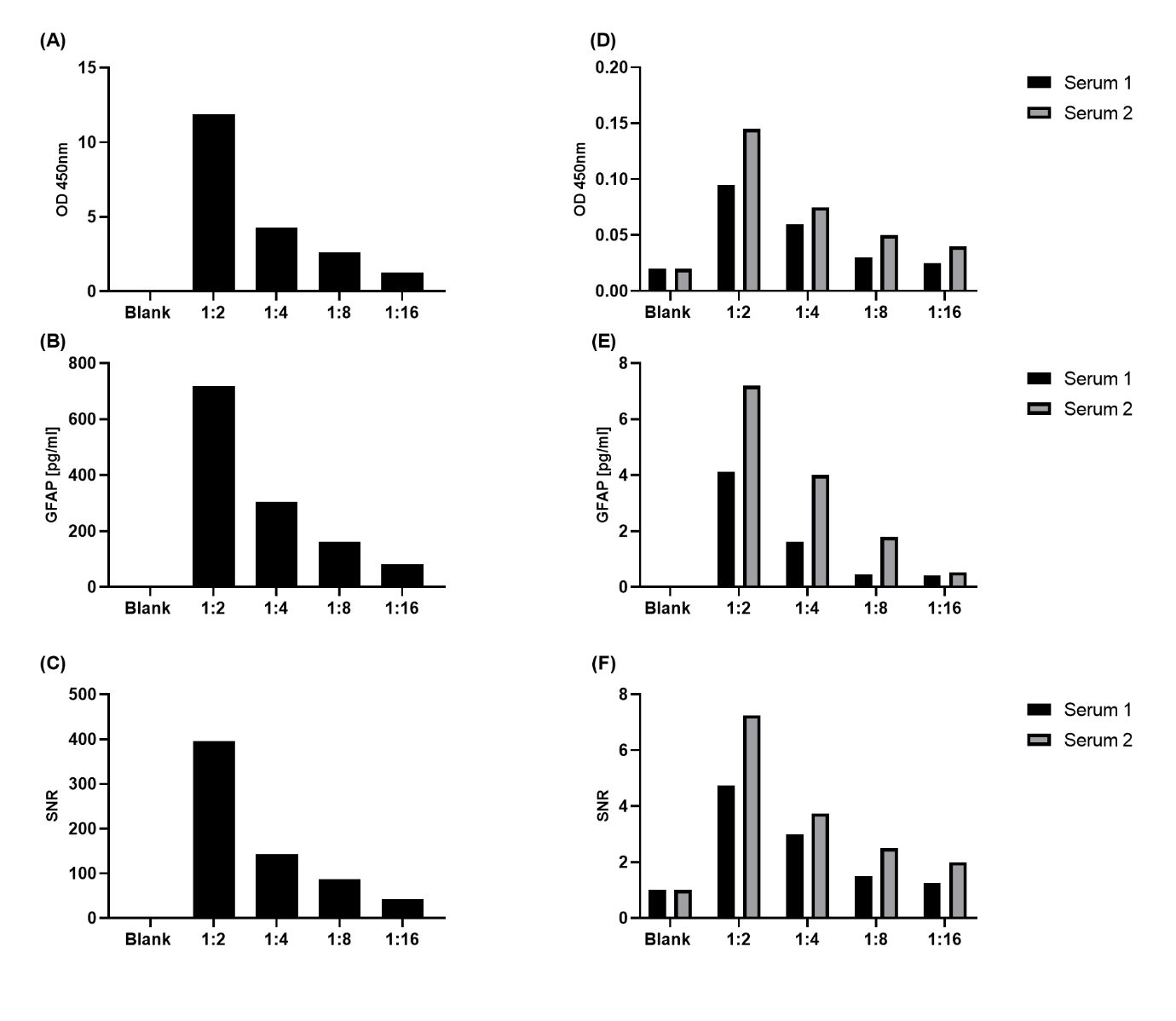
To investigate the parallelism in biological samples, a serial dilution of CSF and two serum samples were prepared, and the parallelism pattern was compared to the standard curve. As can be seen in Figure S1, the signals (Figure S1 A, D), concentrations (Figure S1 B, E), and the signal-to-noise ratio (SNR) values (Figure S1 C, F) decreased when the dilution factor increased in both CSF and serum samples. The serum and CSF samples were control samples with expected low GFAP concentrations. The dynamic range of the assay, based on the standard curve (Figure S2), was considered 3.7 – 2700 pg/mL.

**Figure S1. Parallelism.**

Endogenous GFAP concentrations were analysed in CSF (A, B, C) and serum samples (D, E, F). Column graphs showing the absorbance values (A, D), Concentration (B, E), and SNR values (C, F) obtained from the novel Ella assay to evaluate its ability in detecting GFAP in one CSF and two serum samples. The absorbance signal from the wells of a 48-digoxigenin cartridge, when treated with CSF serial dilutions, can be found in (A), as well as serum serial dilutions in (D). The “Blank” wells were treated with no protein. B and E demonstrate the GFAP concentration (pg/mL) in CSF and serum samples, respectively. C and F show the SNR values of CSF and serum samples, calculated by dividing the absorbance of the wells containing protein by the absorbance of the blank ***
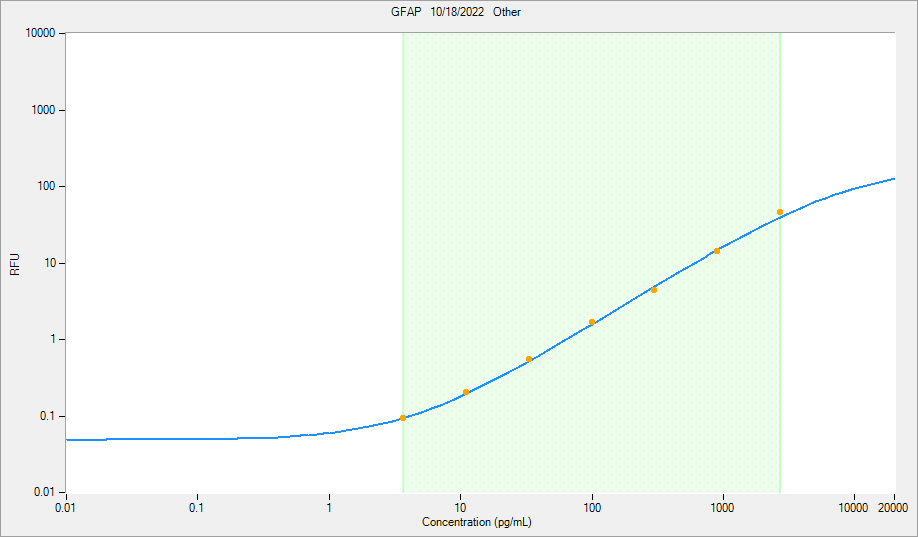
***wells. SNR; Signal-to-Noise Ratio, CSF; Cerebrospinal fluid, OD; optical density.

**Figure S2. Standard curve for calculation of GFAP concentration based on measured signals.**

RFU, Relative fluorescence unit.

To determine the recovery, the dilution series were spiked with 500 pg/mL GFAP recombinant protein Ag10423. The results can be found in Table S1. The parallelism and recovery result considered, the dilution factor of 1:2 and 1:8 was chosen to utilize in further experiments for serum and CSF samples, respectively.

**Table S1**. Spike and recovery results of the serial dilution of CSF and Serum control samples spiked with 500 pg/mL GFAP recombinant protein.

| Sample | Recovery |
| --- | --- |
| CSF 1:2 | 113% |
| CSF 1:4 | 96% |
| CSF 1:8 | 97% |
| Serum 1 1:2 | 86% |
| Serum 1 1:4 | 88% |
| Serum 1 1:8 | 84% |
| Serum 1 1:16 | 93% |
| Serum 2 1:2 | 84% |
| Serum 2 1:4 | 87% |
| Serum 2 1:8 | 88% |
| Serum 2 1:16 | 95% |

CSF, cerebrospinal fluid

**Pre-analytical stability of CSF and serum GFAP**

To assess the GFAP stability in biological samples while storage and handling, CSF and serum samples were used in this experiment. Each sample was divided into 60 µL aliquots. These start samples were referred to as the reference and stored at -80°C. The rest of the aliquots were treated in two temperature conditions (RT, 4°C) for different periods (hours to days). Other aliquots were used to assess the effect of freeze-thaw cycles (FTCs). To compare the concentrations, the reference aliquot concentration was considered to be 100%, and the treated aliquot concentrations were calculated relatively. The obtained results can be found in Figure S3.

**
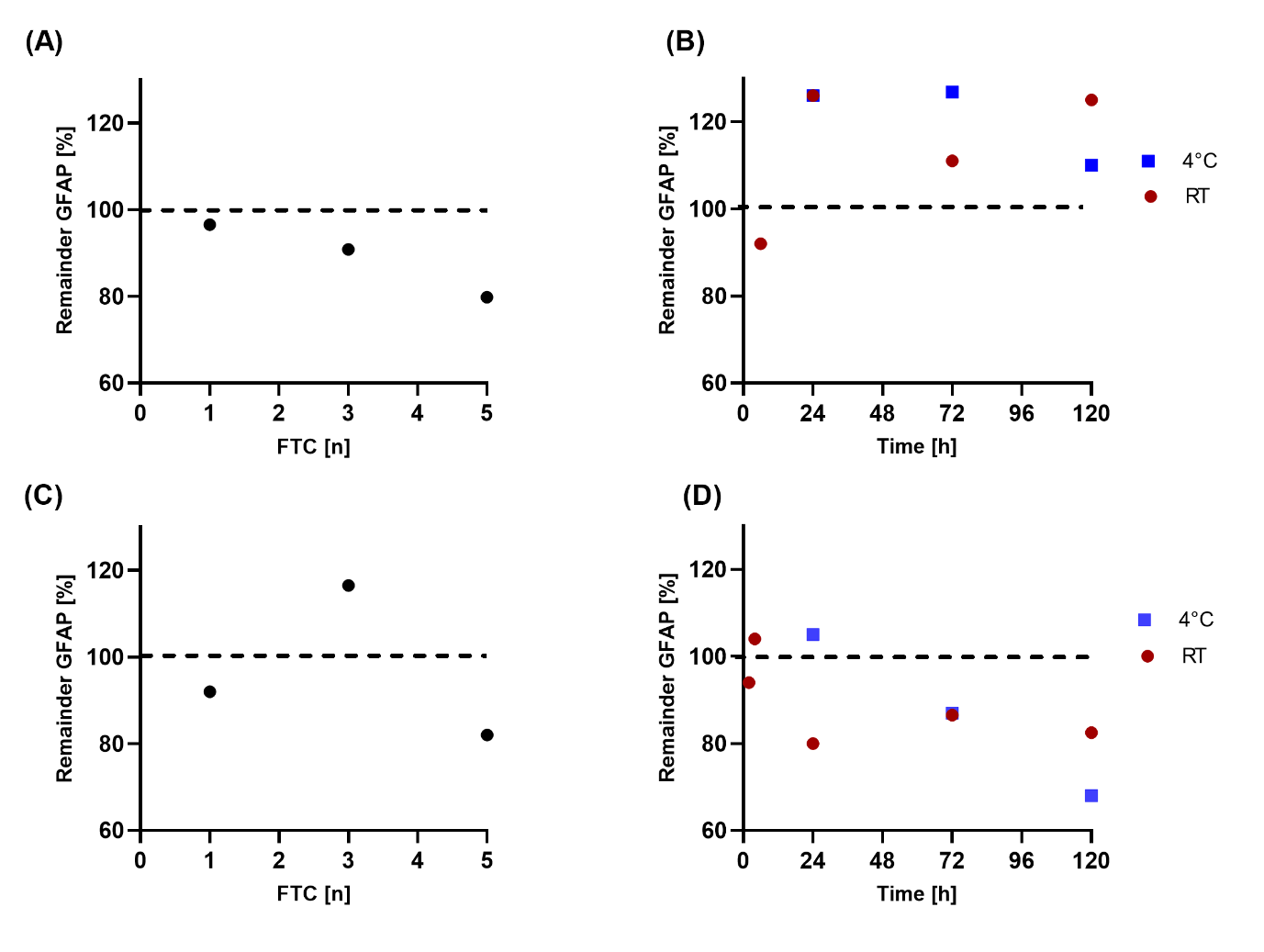
**

**Figure S3. Determination of stability of CSF and serum GFAP.**

(A) CSF GFAP results for experimental freeze/thaw cycles. (B) CSF GFAP results for different experimental storage stability conditions. (C) Serum GFAP results for experimental freeze/thaw cycles. (D) Serum GFAP results for experimental storage stability conditions. Circles demonstrate RT and squares 4 °C results. Symbols represent mean GFAP levels from duplicate measurements, normalized to the reference sample. RT; room temperature, FTC; freeze/thaw cycles, GFAP; Glial fibrillary acidic protein.
